# Operationalizing the CBDR-RC principle in the WHO Pandemic Agreement: from environmental law to pandemic governance

**DOI:** 10.3389/fpubh.2026.1880687

**Published:** 2026-09-30

**Authors:** Junya Lian

**Affiliations:** School of Law, Tongji University, Shanghai, China

**Keywords:** Common but Differentiated Responsibilities and Respective Capabilities, differentiated obligations, global health equity, Pathogen Access and Benefit-Sharing, WHO Pandemic Agreement

## Abstract

**Introduction:**

The World Health Organization (WHO) Pandemic Agreement, adopted in May 2025, represents a major step toward strengthening global pandemic governance after COVID-19. However, the principle of Common but Differentiated Responsibilities and Respective Capabilities (CBDR-RC) was not incorporated into the legally binding provisions of the Agreement, leaving unresolved questions regarding how equity-based obligations should be implemented in future pandemic preparedness and response.

**Methods:**

This study adopts a qualitative legal methodology combining doctrinal legal analysis, comparative doctrinal analysis, and historical-documentary analysis. It examines international legal instruments and jurisprudence, including the 2025 International Court of Justice advisory opinion, WHO Intergovernmental Negotiating Body draft texts from 2021 to 2026, and official submissions and negotiating positions of selected States and negotiating groups.

**Results:**

The analysis identifies three principal findings. First, negotiations over the Pandemic Agreement demonstrate a progressive narrowing of CBDR-RC language, reflecting persistent disagreements between developed and developing countries regarding differentiated obligations. Second, comparative analysis of existing access and benefit-sharing mechanisms shows that equity principles require institutional arrangements rather than voluntary commitments alone. Third, the study proposes a capacity-sensitive framework for the PABS Annex based on three interconnected mechanisms: (1) tiered compliance pathways reflecting national capacities; (2) conditional technology transfer linked to pathogen sharing; and (3) trigger-based emergency financing mechanisms.

**Discussion:**

Operationalizing CBDR-RC within the PABS Annex could transform equity from a general aspiration into a structured governance mechanism for pandemic preparedness. By aligning obligations with capacity, vulnerability, and contribution, the proposed framework could provide regulatory options for improving equitable access to vaccines, therapeutics, and diagnostics while strengthening global health security.

## 1 Introduction

Pandemics including SARS, 2009 H1N1 influenza, Ebola, and COVID-19 have repeatedly exposed critical structural gaps in global health governance—most notably the characterization of resource allocation, fragmentation of disease surveillance, and nationalization of vaccine access (1). In particular, the COVID-19 pandemic, which caused nearly 7 million confirmed deaths globally as of May 2023 (2), is widely recognized as one of the worst public health catastrophes of the modern era, and the stark inequalities in COVID-19 vaccine distribution lay bare deep-seated structural inequities within the global health governance system (3). High-income countries procured more vaccine doses than their domestic needs required, while the majority of low-income countries were unable to achieve minimal population-wide vaccination coverage (4). By September 2022, developed countries secured vaccine coverage of 75%, while many least developed countries (LDCs) recorded vaccination rates as low as 39% (5). The existing international public health legal regime, anchored in the International Health Regulations (2005) (IHR) (6) and supplemented by fragmented regional cooperation frameworks and sector-specific treaties, remains structurally inadequate to address these inequities (7). Systemic inequities prioritizing commercial interests, failed soft-law norms, and the lack of an equity-centered binding pandemic instrument demand a binding health rights framework (3).

In response, the World Health Assembly (WHA) of the World Health Organization (WHO), which adopts key international legal instruments governing pandemic prevention, preparedness and response (PPPR) (8), launched a comprehensive reform of international health law via a two-track intergovernmental negotiation framework which is centered on the principles of solidarity and equity (9). To be specific, it established an INB to draft and negotiate a new comprehensive WHO convention, agreement, or other international instrument on PPPR, and initiated targeted amendments to the IHR which merely provides a legal framework for pandemic preparedness, in order to modernize the existing legal framework governing PPPR (6). Notably, the WHO Pandemic Agreement was formally adopted by the 78th WHA on 20 May 2025 (10), and the IHR amendments also entered into force in September 2025 (11). The next step in operationalizing the WHO Pandemic Agreement is the negotiation of detailed rules for the Pathogen Access and Benefit-Sharing (PABS) System—a core pillar of the Agreement, which will be enshrined in a binding annex to the Agreement (12).

Central to the intergovernmental negotiations of the WHO Pandemic Agreement was the prolonged debate over the formal incorporation of the principle of Common but Differentiated Responsibilities and Respective Capabilities (CBDR-RC) (13). CBDR-RC is a principle of international law that seeks to reconcile the universal nature of collective obligations with substantive equality by requiring States to share common responsibilities while assuming differentiated duties according to their historical contributions and respective capabilities (14). Led by the Intergovernmental Negotiating Body (INB) Equity Group, developing States advocated for the explicit enshrinement of binding CBDR-RC provisions in the WHO Pandemic Agreement. They emphasized that the operationalization of this principle requires mandatory measures to ensure the pharmaceutical sector contributes fully to global pandemic governance, including through the sharing of intellectual property, non-voluntary binding technology transfer to developing States, and the equitable distribution of monetary benefits to strengthen developing States’ capacities for pandemic preparedness, surveillance, and rapid emergency response (15). In contrast, other States, including several high-income developed States, contended that the CBDR-RC principle was not an established, legally binding general principle of international health law. They further argued that the principle originated in the climate change regime and had no general applicability to global health, and that its normative core was already sufficiently encapsulated within the Pandemic Agreement’s overarching guiding principles of equity and solidarity (13).

The persistent differences among States over the formal incorporation of the CBDR-RC principle in the WHO Pandemic Agreement is rooted in two interrelated structural drivers. First, the negotiation process of the WHO Pandemic Agreement was significantly shaped by the commercial interests of manufacturers of pandemic-related health products, which amplified the underlying tension between the competing priorities of developed and developing States (13). Developed States had long secured a dominant position in the research and development (R&D), manufacturing, regulatory oversight, and global distribution of pandemic vaccines by virtue of their accumulated technological advantages, financial capacity, and complete industrial chains. In contrast, developing States, constrained by chronic structural gaps in funding and core technologies, face inherent limitations in fulfilling the same level of uniform, undifferentiated obligations as developed States. This structural imbalance formed the core normative basis for developing States’ advocacy for a binding differentiated responsibility framework aligned with respective national capabilities, as operationalized through the CBDR-RC principle (16). Second, this North–South divergence stems from a normative dispute over the legal integration of the CBDR-RC principle into binding international health law. While Article 3.4 of the WHO Pandemic Agreement enshrines equity as a principle of PPPR, it makes no explicit reference to the CBDR-RC principle. The normative anchoring of the CBDR-RC principle in the global pandemic governance framework thus remains unresolved. This unresolved status echoes broader debates regarding the gap between aspirational equity language and enforceable legal obligations in international health instruments (3).

A critical preliminary question must be addressed: can the CBDR-RC principle originating in international environmental law legitimately be applied to global health governance? We argue that it can, for three interconnected reasons. First, both climate change and its cascading public health consequences—including the emergence and spread of climate-related diseases and amplified pandemic risks—fall within the scope of global common concerns requiring collective action. The International Court of Justice (ICJ) confirmed in its 23 July 2025 advisory opinion (ICJ 2025 Advisory Opinion) that climate change itself constitutes a global common concern, and its inherent transboundary public health spillovers are an inseparable and foreseeable dimension of this concern, thus equally subject to the principle of collective international responsibility (17). Second, both climate change and global health crises exhibit colonial-rooted structural asymmetries in historical responsibility and current capacity: just as industrialized nations contributed disproportionately to cumulative greenhouse gas emissions, high-income countries have historically extracted disproportionate benefits from global pathogen surveillance networks and biomedical innovation systems (direct legacies of 19th-century colonial public health regimes) while failing to advance equitable access to life-saving health technologies, and currently possess vastly superior public health emergency response capabilities and resource mobilization capacities compared to low- and middle-income countries (18). Third, the WHO Commission on Social Determinants of Health framework demonstrates that avoidable health inequities are not accidental, but substantially shaped by the unequal distribution of power, income, and resources across the global political and economic order—conditions in which people are born, grow, live, work, and age (19). Crucially, many of these structural inequities in low- and middle-income countries are rooted in historical colonial legacies that continue to perpetuate systemic disadvantage. This structural injustice creates a clear ethical imperative for differentiated national and global obligations to redress these harms and advance health equity (19). These three parallel logics—collective responsibility for transboundary global common concerns, historical accountability paired with differentiated current capacity, and ethical obligation to redress avoidable structural inequities—provide a robust normative foundation for extending the CBDR-RC principle to global health governance. While existing literature has extensively examined the normative aspirations of equity in global health, a critical knowledge gap remains regarding how abstract legal principles—specifically the CBDR-RC principle—can be translated into enforceable, binding legal mechanisms within post-COVID global health governance architecture. Previous studies predominantly treat CBDR-RC as an environmental law doctrine, failing to bridge the gap between international legal theory and the operational mechanics of public health emergency response, global health security, and biomanufacturing equity. Against this background, this study asks a central question: how can the CBDR-RC principle, originally developed within international environmental law, be legally operationalized within the post-COVID global health governance framework to create enforceable and capacity-sensitive obligations for pandemic prevention, preparedness and response? This study fills this gap by examining the concrete legal-policy pathways through which CBDR-RC can be institutionalized to address the structural determinants of health inequities.

Against this backdrop, this paper addresses three sequential research questions: (1) What is the theoretical and legal basis for applying the CBDR-RC principle in global pandemic governance? (2) How was the CBDR-RC principle contested during the INB negotiations, and what were the key fault lines between developed and developing States? (3) How can the CBDR-RC principle be concretely operationalized within the PABS Annex to advance global health equity and strengthen health system resilience? Drawing on a systematic analysis of successive INB treaty drafts, official position statements and submissions from key negotiating groups and States, including the Group for Equity, the Africa Group, the G77 and China, the European Union, and the United States, and the final adopted text of the WHO Pandemic Agreement, this study develops a structured framework for embedding the CBDR-RC principle in the PABS Annex. The negotiating groups and States examined in this study were selected purposively rather than exhaustively to reflect divergent approaches to differentiation. These actors were included because their official submissions provide identifiable and contrasting positions on differentiation, equity, capacity, vulnerability, contribution, and benefit-sharing. Specifically, it argues that the CBDR-RC principle can be operationalized through three interdependent mechanisms: tiered obligations calibrated to national capacity, conditional technology transfer provisions, and trigger-based financing arrangements. By transforming high-level equity rhetoric into actionable legal commitments, this framework aims to safeguard health equity, enhance global health security, and optimize inter-sectoral health governance for future health emergencies.

To address the research questions, this study adopts a multi-tiered qualitative legal methodology, primarily combining doctrinal legal analysis, comparative doctrinal analysis, and historical-documentary analysis. These methods are selected because the research questions concern not only the legal characterization and normative content of the CBDR-RC principle, but also its possible institutional transformation from an environmental law concept into an operational framework for post-COVID global health governance. Doctrinal legal analysis is employed to examine the normative scope, legal status, interpretive principles, and evolutionary trajectory of CBDR-RC across international legal instruments. Comparative doctrinal analysis is used to assess how differentiated responsibility mechanisms operate across distinct international regimes, particularly by comparing environmental legal frameworks (e.g., the UN Framework Convention on Climate Change (UNFCCC) and the Paris Agreement) with global health governance instruments (e.g., the Framework Convention on Tobacco Control (FCTC) and the IHR). In addition, historical and archival documentary analysis is conducted to trace the textual evolution of pandemic treaty negotiations, identify competing state positions, and examine how equity-related obligations have been negotiated within the INB process leading to the adoption of the WHO Pandemic Agreement and the ongoing negotiations concerning the PABS Annex. The comparative and historical findings are then synthesized around five analytical dimensions—legal status, basis for differentiation, institutional mechanisms, legal enforceability, and enabling support—and four recurring operational criteria—capacity, vulnerability, contribution, and implementation responsibility—which inform the proposed framework for operationalizing CBDR-RC in pandemic governance.

The primary legal and policy materials examined in this study include: (1) international treaties, declarations, and soft-law instruments relevant to differential obligations and global health governance, including the Rio Declaration on Environment and Development of 1992 (Rio Declaration), UNFCCC, Kyoto Protocol, Paris Agreement, the United Nations Convention on the Law of the Sea (UNCLOS), FCTC, IHR 2005, and the Alma-Ata Declaration; (2) official WHO and INB negotiation documents from 2021 to 2026, including the Working Draft (2022), Conceptual Zero Draft (2022), Zero Draft (2023), Bureau’s Texts (2023–2024), New Draft Negotiating Text (2024), and the adopted WHO Pandemic Agreement (2025); (3) relevant international jurisprudence, particularly the advisory opinion of the ICJ issued on 23 July 2025; and (4) official statements, submissions, and negotiating positions from state groups and actors selected to reflect divergent approaches to differentiation, including the Group for Equity, the Africa Group, the G77 and China, the European Union, and the United States. Secondary sources consist primarily of peer-reviewed academic literature in the fields of international law, global health governance, health equity, and international development.

A structured legal document identification strategy was employed to locate and select relevant primary and secondary materials. Searches were conducted through major legal, biomedical, and interdisciplinary databases, mainly including HeinOnline, Web of Science, PubMed, LexisNexis, and official WHO, INB, and World Health Assembly document archives. The historical scope of the research extends from 1992, when CBDR-RC was formally articulated in the Rio Declaration. For the contemporary analysis, the documentary and negotiating evidence reviewed extends through 20 July 2026. This temporal scope allows the study to examine both the historical development of the principle and its contemporary application in pandemic governance. Search terms included combinations of keywords such as “Common but Differentiated Responsibilities,” “CBDR-RC,” “Pandemic Agreement,” “Pathogen Access and Benefit-Sharing,” “PABS,” “technology transfer,” “benefit-sharing,” “pandemic financing,” “International Health Regulations,” and “Global Health Equity.” Materials were selected based on the following inclusion criteria: (i) official treaty texts, negotiation documents, and institutional records directly related to CBDR-RC or pandemic governance; (ii) binding international instruments, judicial decisions, or authoritative legal materials addressing differentiated obligations and state responsibility; and (iii) peer-reviewed academic analyses examining equity, treaty design, and global health governance. For negotiation materials, documents were included when they contained substantive treaty language, formal proposals, or identifiable negotiating positions relevant to differentiation, equity, or PABS. Non-official commentaries lacking primary-source support, duplicate materials, and non-English documents without reliable official translations were excluded.

Methodologically, this study acknowledges the inherent limitations of a predominantly doctrinal and historical legal approach. Doctrinal analysis provides detailed insight into textual interpretation, normative structures, and institutional design, but it cannot fully capture broader political economy dynamics, geopolitical bargaining processes, or the actual implementation and compliance behavior of states after legal obligations are adopted. Similarly, because the PABS Annex negotiations remain an evolving process, the analysis is based on regulatory, institutional, and negotiating developments documented and publicly available through 20 July 2026. It therefore incorporates the outcome of the seventh Intergovernmental Working Group on the WHO Pandemic Agreement (IGWG) meeting held from 6 to 17 July 2026, while not incorporating developments from the eighth IGWG meeting scheduled for 14–18 September 2026. To address these limitations, the study contextualizes doctrinal findings through comparative analysis across international regimes and incorporates insights from official negotiating positions, institutional documents, and existing scholarship on pandemic inequities. The proposed framework is consequently derived from the convergence and divergence identified across these materials, rather than from quantitative measurement or empirical claims about state behavior. Accordingly, the conclusions drawn are directed primarily toward assessing the legal feasibility and institutional design of CBDR-RC operationalization rather than predicting future political outcomes or measuring post-adoption compliance empirically.

## 2 Theoretical foundations and normative evolution of the CBDR-RC principle: from environmental law to global health governance

### 2.1 The genesis of the CBDR-RC principle

The principle of CBDR-RC, while most prominently codified in the 1992 Rio Declaration at Principle 7 (20), has deeper roots. Provisions embodying the concept of differential treatment predate the Rio Declaration and can be found in earlier international legal instruments, including the Constitution of the International Labour Organization, the General Agreement on Tariffs and Trade, and the UNCLOS—specifically in the chapter establishing the legal regime governing the international seabed area (9). Its most recent and high-profile application is in the field of international climate change law, where the Paris Agreement recalls the CBDR-RC principle among its guiding principles (13).

Although the principle of CBDR-RC has gone through considerable upheavals, the principle has been progressively recognized in the international legal framework (13). Especially, the principle of CBDR-RC has been a core guiding principle for the implementation of the climate change treaties (21). To be specific, the Intergovernmental Panel on Climate Change (IPCC) recognized in its 1990 First Assessment Report that “[a] major part of emissions affecting the atmosphere at present originates in industrialized countries” (22). This finding was endorsed in the principle of CBDR-RC in the 1992 Rio Declaration, mainly in Principles 6 and 7. The principle of CBDR-RC was first codified in Article 3.1 of 1992 UNFCCC to address climate justice (23). This marked a turning point in global environmental governance, as for the first time, a political consensus was reached between developed and developing countries, that developed countries admitted their historical responsibilities, and agreed to assume higher obligations to combat environmental and climate change challenges (13). Later, the Kyoto Protocol, the first protocol to the UNFCCC, further develops the principle of CBDR-RC in Article 10 in 1997. And the Paris Agreement, a separate treaty instrument adopted under the UNFCCC framework, also recognizes CBDR-RC in Article 2.2 and Article 4.3. The core normative function of the CBDR-RC principle in international environmental law is to frame environmental protection as a universal obligation of all states. However, industrialized nations historically contributed disproportionately to cumulative greenhouse gas emissions during their unregulated industrialization processes, through which they achieved rapid economic development and accumulated substantial global wealth. This historical asymmetry creates a compelling normative basis for these countries to bear correspondingly greater mitigation and adaptation obligations in addressing the global climate crisis (24).

From a normative standpoint, the CBDR-RC framework may appear to contradict the foundational principle that “as Members of the community of nations they are equals, whatever differences between them may otherwise exist” (25), which is a well-established principle of international law, particularly reflected in the United Nations Charter. However, the principle is more appropriately interpreted as a normative vehicle for advancing substantive equality between states: it explicitly recognizes and redresses structural cross-national disparities through non-reciprocal, context-specific obligations, thereby advancing a fairer, more equitable application of international law. In practice, such differential operationalization translates into concrete institutional arrangements, including extended compliance periods, differentiated legal commitments, and exemptions from certain responsibilities that explicitly take into account national socio-economic situations, developmental capacities, and structural vulnerabilities. In this sense, it constitutes a concrete manifestation of the general principle of equity in international law. This is because the core normative function of the CBDR-RC principle in international environmental law is to frame environmental protection as a universal obligation of all states, while simultaneously recognizing that industrialized nations historically contributed disproportionately to cumulative greenhouse gas emissions during their unregulated industrialization processes—a historical asymmetry that created a compelling normative basis for these countries to bear correspondingly greater obligations (13). Whereas general equity mechanisms in international law often function through reciprocal flexibilities or narrow exceptions, the CBDR-RC principle operates through a framework of distributive justice (26). It reallocates global responsibilities and resource flows based on structural capacity and historical contribution rather than strict formal reciprocity, thereby operationalizing the broader international law imperatives of fairness and solidarity (27).

### 2.2 From environmental law to global health: diffusion and adaptation

Although the principle of CBDR-RC originated in international environmental law, functionally related forms of differentiation have also emerged in other areas of international law. For example, the principle of CBDR-RC can be found in the World Trade Organization (WTO) Agreements in the form of “special and differential treatment” provisions which grant developing countries flexible commitments and technical assistance, and its spirit is drawn in Articles 203 and 278 of the UNCLOS (13). These mechanisms should not be equated doctrinally with CBDR-RC, but they demonstrate that differentiated responsibilities and capacity-sensitive treatment are not confined to environmental law. Crucially, counter to assertions by developed states that CBDR-RC is foreign to global health governance, the FCTC—the only other legally binding health treaty adopted under the auspices of the WHO prior to the Pandemic Agreement—contains provisions that reflect elements of differentiated responsibility. This existing precedent challenges the claim that differentiated responsibility lacks institutional standing in binding international health law. Specifically, the FCTC operationalizes CBDR-RC through binding differential treatment obligations, including targeted technical and financial assistance (Article 4.6), cooperation to strengthen domestic capacities (Article 22.1), dedicated health financing (Article 26.3), and the prioritized mobilization of financial and technical resources for developing nations (Article 26.5(a)). The FCTC therefore demonstrates that differentiated responsibilities and capacity-sensitive obligations already have an institutional basis in binding international health law, even though it does not expressly formulate these provisions in terms of CBDR-RC (13).

A particularly important jurisprudential reference is the ICJ 2025 Advisory Opinion. The Opinion provides significant clarification of the legal role of CBDR-RC, but its reasoning must be understood within the specific context of climate change and international environmental law. In paragraph 148, the Court described CBDR-RC as “a core guiding principle for the implementation of the climate change treaties” and explained that it “reflects the need to distribute equitably the burdens” of climate-related obligations, taking into account States’ historical and current contributions and their different capabilities and national circumstances (21). The Court further explained in paragraph 151 that CBDR-RC is “a manifestation of the principle of equity” and guides the interpretation of obligations under international environmental law (28). Importantly, however, the Court also stated that CBDR-RC “does not establish new obligations” but is relevant to treaty interpretation and the determination of customary rules relating to the environment (28). The operative part of the Opinion likewise applies CBDR-RC in the context of States’ obligations concerning the climate system and other aspects of the environment. The Opinion thus recognizes and elaborates upon the legal relevance of equity, differentiated responsibility, historical and current contributions, capabilities, and national circumstances within the climate-change regime, but it does not establish CBDR-RC as an independently binding principle applicable across all fields of international law. Nor does it expressly extend the principle to global health governance.

The relevance of the ICJ Opinion to pandemic governance is therefore advanced in this article as a legal and normative argument, rather than as a conclusion established by the Court. The proposed application of this reasoning to pandemic governance rests on an interpretive analogy between climate governance and pandemic governance. Both involve transboundary risks, collective-action problems, substantial differences in States’ capacities, and distributive questions concerning the allocation of responsibilities, resources, and benefits. These structural parallels do not make the two legal regimes legally interchangeable, nor do they establish that CBDR-RC automatically applies to pandemic governance. They nevertheless provide a basis for examining whether the equity-oriented and capacity-sensitive reasoning articulated by the Court can be adapted, through treaty design and institutional practice, to pandemic governance and, in particular, to the PABS system. Accordingly, this article treats the 2025 ICJ Advisory Opinion as a jurisprudential reference that supports, but does not itself establish, the proposed adaptation of CBDR-RC to global health. The argument developed below is therefore the article’s own legal and normative proposal concerning how CBDR-RC-informed differentiation might be operationalized in pandemic governance, rather than a claim that the ICJ has already established such an obligation under international health law.

### 2.3 Core tenets of the CBDR-RC principle

The principle of CBDR-RC acknowledges, on the one hand, the historical responsibility of certain states and, on the other, that the measures which can be expected from all states with respect to addressing the common challenge are not the same (21). The core tenets of the CBDR-RC principle include two components: responsibility and capacity. State responsibility reflects the need to distribute equitably the burdens of the obligations in respect of global common concern by taking all states’ historical and current contributions to the current challenges into consideration. State capacity is determined by divergent national circumstances, most notably varying levels of economic and social development, which necessitates differentiating each country’s “fair share” of common responsibilities based on a set of applicable equity benchmarks (29).

States have reached a consensus concerning the commonness of responsibility based on the recognition of addressing a challenge posed by a common concern to humankind (16). This collective responsibility framework has deep roots in international health law. For instance, the 1978 Alma-Ata Declaration explicitly recognized that stark health inequalities between developed and developing countries constitute a common concern for all states (30), a position reinforced by the UN Committee on Economic, Social and Cultural Rights in General Comment No. 14, which clarifies that states bear a collective responsibility to address cross-border communicable disease threats (31). However, the intense debate centers on the differentiated allocation of responsibilities under the CBDR-RC principle. Key questions in this regard include the rationale for establishing such differentiated treatment and the specific ways in which such differentiation should be implemented in line with this principle (16).

The answer to the first question might be based on the equity and fairness in international law. It means that those who have contributed more to the problem bear greater obligations to alleviate it, while those with greater capacity also bear a greater share of the burden. In its 2025 Advisory Opinion, the ICJ characterized CBDR-RC as “a manifestation of the principle of equity” and recognized its relevance to the interpretation of obligations under international environmental law (28). The harmful effects of environmental degradation have been accumulating for two or three centuries since the advent of the Industrial Revolution. The historical responsibilities of developed countries have been affirmed in the negotiations of international environmental legal instruments, like the UNFCCC and the Kyoto Protocol (16). Similarly, colonial legacies shape historical inequity in the PPPR. Western industrial exploitation and colonialism created resource disparities and vulnerable health systems, justifying CBDR-RC’s fairness principle to allocate differentiated responsibilities (24). Besides, General Comment No. 14 elaborates that “economically developed States have a special responsibility and interest to assist the poorer developing States” in realizing the right to the highest attainable standard of health (31).

The second question pertains to the types of priorities that developing countries should have vis-à-vis developed countries. Firstly, developing countries are granted an extension of the timeframe to fulfil their commitments, as exemplified by the Montreal Protocol. Secondly, certain substantive obligations are exclusively assigned to developed countries, as exemplified by the Kyoto Protocol. Thirdly, there are provisions regarding non-compliance that are more lenient for developing countries, as exemplified by the Cartagena Protocol on Biosafety to the Convention on Biological Diversity of 2000. Fourthly, developed countries often bear substantive obligations to assist developing countries in the implementation process. In summary, the application of CBDR-RC yields two primary outcomes: developing countries should receive differentiated treatment regarding core obligations in international health law just as international environmental agreements, such as extended compliance periods and exemptions from certain responsibilities that take into account their socio-economic situation, their developmental needs and their vulnerabilities; this differentiation does not exclude the responsibility of developing countries but their performance depends on the support like technology transfer and financial support provided by developed States (32).

Another contentious debate regards the legal status of the CBDR-RC principle as a rule of international law. In particular, developed countries argue that this principle remains a principle of international environmental law employed within regulatory frameworks to guide the interpretation and implementation of obligations (16). However, as discussed above, the ICJ in its advisory opinion in 2025 did not characterize that the principle of CBDR-RC as a general principle of international law and it does not establish new obligations. To facilitate interdisciplinary understanding and avoid an exclusively narrative presentation, Table 1 provides a comparative overview of how differential treatment mechanisms and CBDR-RC-related approaches have evolved across different international legal regimes, and how these experiences inform the debate on global health equity.

**Table 1:** Comparative analysis of CBDR-RC and differential treatment mechanisms across international governance regimes.

Governance regime	Key legal instrument/case	Year	Main legal outcome/operational mechanism	Relevance to global health equity
International Environmental Law	Rio Declaration Principle 7; UNFCCC	1992	Formally articulated the CBDR-RC principle and established that global environmental obligations should be implemented through differentiated approaches reflecting historical contribution, developmental circumstances, and financial and technological capacities. The UNFCCC further operationalized differentiation through different commitments for Annex I and non-Annex I States.	Provides the foundational legal rationale that collective action addressing global challenges may require capacity-sensitive obligations to achieve substantive equality.
Climate Governance	Kyoto Protocol; Paris Agreement	1997; 2015	The Kyoto Protocol adopted a relatively categorical differentiation model, while the Paris Agreement introduced a more flexible approach based on nationally determined contributions and CBDR-RC “in light of different national circumstances.”	Demonstrates the evolution of differentiated responsibility from fixed classifications toward adaptive and capacity-sensitive implementation mechanisms.
International Law	ICJ Advisory Opinion on Climate Change	2025	Recognized CBDR-RC as a core guiding principle for implementation of climate-change treaties; linked it to equity, historical and current contributions, capabilities and national circumstances; held that it guides interpretation of environmental obligations without itself establishing new obligations.	Provides a jurisprudential reference for examining capacity- and equity-sensitive differentiation in other transboundary governance regimes; its application to global health is proposed by this article rather than established by the ICJ.
International Trade and Oceans Governance	WTO Agreements on Special and Differential Treatment; UNCLOS Articles 202–203	1982; 1994	Established differentiated treatment mechanisms through technical assistance, capacity building, and preferential arrangements for developing States.	Demonstrates that capacity-based differentiation is a recurring institutional feature of international governance beyond environmental law.
Global Health Law	FCTC	2003	Incorporated binding cooperation obligations, including technical assistance, capacity building, and international support mechanisms for developing States.	Provides a direct precedent within WHO treaty practice for translating equity principles into operational public health obligations.
Genetic Resources and Health Governance	The International Treaty on Plant Genetic Resources for Food and Agriculture (ITPGRFA); PIP Framework	2001; 2011	Established access and benefit-sharing (ABS) mechanisms linking access to biological materials, information sharing, and the equitable distribution of resulting benefits.	Provides institutional lessons for designing the PABS Annex by addressing structural asymmetries between resource providers and users in global health governance.

CBDR-RC and differentiated treatment mechanisms are not identical legal concepts. This table compares related institutional approaches through which international regimes accommodate differences in capacity, contribution, and vulnerability.

## 3 The contested status of the CBDR-RC principle: state positions and textual evolution in the WHO Pandemic Agreement negotiations

The CBDR-RC principle was a core, highly contentious issue throughout the negotiations of the WHO Pandemic Agreement (33). Member States from developing countries consistently advocated for the explicit, binding incorporation of the CBDR-RC principle into the treaty text, while developed Member States opposed its formal enshrinement as an independent guiding principle, arguing that its normative core was already addressed by the Agreement’s overarching principles of equity and international solidarity. This deep-seated North–South divide over the CBDR-RC principle was a defining feature of the WHO Pandemic Agreement negotiations, reflecting different normative views on equity, differentiated obligations, and state responsibility in global pandemic governance.

### 3.1 Divergent state positions on embedding the CBDR-RC principle in the pandemic agreement

#### 3.1.1 Strong advocates for explicit CBDR-RC provisions

A broad coalition of developing countries has consistently advocated for the formal inclusion of the CBDR-RC principle in the WHO Pandemic Agreement to ensure global equity in health and to strengthen PPPR. Although a group of 47 African countries, along with Bangladesh, Brazil and Paraguay called for the inclusion of the CBDR-RC principle, the INB Bureau altered the terminology, as originally articulated in the UNFCCC, to “shared but differentiated responsibilities and capabilities” (SBDRC) in the Working Draft (34) for the INB’s second meeting (35). Crucially, the words “common” and “respective” from the established CBDR-RC principle do not appear in Article 4.9 (36), a modification that compromised the jurisprudential application of this principle and raised several concerns, especially for developing countries (35). Specifically, Bangladesh, Malaysia, Pakistan and Namibia explicitly called for the normative, internationally recognized CBDR-RC terminology to replace the revised phrasing of SBDRC (35). In justifying the incorporation of the CBDR-RC principle, Brazil pointed to stark cross-national disparities in medical product hoarding and stockpiling capacities, while Paraguay clarified that the principle aims to facilitate a coordinated global pandemic emergency response rather than impose fixed national responsibilities (35). Namibia went further, emphasizing that operationalizing the CBDR-RC principle requires binding obligations on the pharmaceutical industry, including intellectual property sharing and technology transfer to developing countries, in order to advance global health equity (35). Bangladesh underscored the CBDR-RC principle as the most critical treaty element for developing countries, formally demanding a correction of the erroneous draft phrasing, and warning that excluding the principle would severely constrain developing countries’ implementation of the One Health Approach (35).

This advocacy persisted. At the INB-3 meeting held in December 2022, developing states including Bangladesh, South Africa (37), Brazil (38) and China (39) advocated for the inclusion of the CBDR-RC principle in the Working Draft, emphasizing its link to equity, rejecting one-size-fits-all solutions, and calling on developed countries to support developing nations (1). The momentum built further. At the INB-5 meeting in June 2023, the Group for Equity—a cross-regional coalition of 29 developing WHO Member States—first formally called for concrete, enforceable provisions to fully operationalize equity in the binding pandemic treaty (40). In an October 2023 pre-INB-7 statement (41), the group criticised the Working Draft’s failure to advance equity, urged the adoption of legally binding commitments on core equity-related articles (41), and reaffirmed the CBDR-RC principle as a foundational principle for an equitable global pandemic accord (41).

The negotiations advanced at the INB-8 meeting in February and March 2024, where the INB Bureau was formally mandated to develop a revised draft negotiating text to facilitate text-based negotiations at the 9th INB session (42). Throughout the session, developing country coalitions consistently centered the operationalisation of equity as the non-negotiable core of the proposed pandemic accord. The Africa Group, representing 47 Member States highlighted the critical lack of consensus on Article 12 concerning the PABS and prioritized sustainable local production of medical countermeasures, technology transfer to developing countries, and adequate, solidarity-based financial resourcing, while rejecting unjust additional obligations on nations still grappling with COVID-19 vaccination inequities (42). Concurrently, the Group for Equity outlined six foundational elements for the legally binding instrument: obligations aligned with the CBDR-RC principle and proportionate to national capacities; an equitable, transparent PABS system; binding normative standards for technology transfer and licensing; a predictable, inclusive sustainable financing mechanism; a participatory, accountable governance framework; and universal, barrier-free operability of the accord. Other developing states including India, Bangladesh, Brazil, China and South Africa reiterated the centrality of equity, solidarity and the CBDR-RC principle, demanding these concepts be translated into enforceable provisions (42).

#### 3.1.2 Opponents to the incorporation of the CBDR-RC principle

Developed countries oppose the CBDR-RC principle, deeming it a concept borrowed from other international legal regimes such as climate change and therefore unfit for global health, yet they simultaneously invoke its equity and solidarity components while pushing to mainstream the cross-regime One Health Approach in the WHO Pandemic Agreement (35). At the INB-2 meeting, developed states including Monaco, the United States, New Zealand, Australia, the United Kingdom, and the European Union formally expressed reservations about the explicit inclusion of the CBDR-RC principle in the Working Draft. They uniformly argued that the principle, originating from the UNFCCC and other international legal regimes, is contextually inappropriate for global health governance, contending that its core equity objectives could be realised through operative provisions and principles such as equity and solidarity without explicit textual embedding (35). The INB Bureau aligned with this stance, noting that it may fine-tune or remove the principle from the upcoming Conceptual Zero Draft (35).

This opposition intensified at subsequent sessions. At the INB-3 meeting, the United States claimed that it does not recognize the CBDR-RC as a principle and does not consider this concept appropriate in the context of PPPR, given that the WHO Constitution generally calls for cooperation, investment and action by all countries (43). A similar position was taken by the United Kingdom, which favoured merely considering the special needs of certain countries (44), by Australia, which stated that it did not support the direct application of the CBDR-RC principle in the health context (45), and by Canada, which recommended removing the principle of CBDR-RC from the Working Draft (46). In the same vein, the European Union (47) and Norway (48) also raised some general concerns regarding this issue.

At the INB-9 session in March 2024, opposition from developed states crystallised. Developed countries, including the United States, Japan, Australia, the United Kingdom, Norway, and the European Union formally opposed enshrining both the CBDR-RC principle and the concept of “solidarity” in Article 3.5 of the negotiating text. Australia reiterated its position that CBDR-RC belongs exclusively to environmental law and is therefore inapplicable to global health, while Japan stated that it would never accept the principle in international health law. The United States and New Zealand pushed to delete all references to “solidarity” in Article 3.5, a proposal that was unanimously rejected by 70 developing countries. Furthermore, developed states sought to dilute provisions requiring financial and technical support to developing countries. For instance, the United State and the European Union rejected binding technology transfer, equitable benefit-sharing, and an accountable implementation financing mechanism, potentially limiting developing countries’ equity priorities (49).

### 3.2 From political rhetoric to legal text: the framing of the CBDR-RC principle in successive drafts

The draft WHO Pandemic Agreement was developed over 20 sessions of the INB, held between February 2022 and April 2025, amid often tense negotiations marked by deep North–South divisions (50). The contested status of the CBDR-RC principle is reflected in successive negotiating texts, which reveal a clear pattern: consistent resistance from developed countries and the consequent, systemic dilution of its normative substance (51). The Working Draft of 2022 does not contain the CBDR-RC principle in its proper form. By contrast, the Zero Draft of 2023 did incorporate the principle explicitly. However, this principle was subsequently downgraded to merely one of several options in the later revised Bureau’s Text before being entirely removed from the final negotiating text and ultimately excluded from the adopted WHO Pandemic Agreement.

#### 3.2.1 The working draft (2022)

On 13 July 2022, the INB Bureau released the first formal Working Draft of the WHO Pandemic Agreement for consideration at the 2nd INB meeting (34). While the preamble explicitly acknowledges that “pandemic prevention, preparedness and response at all levels and particularly in developing countries require sufficient financial and technical resources” (34), the Working Draft appears to depart from established international law by replacing the universally recognized principle of CBDR-RC with the novel terminology of SBDRC in Article 4.9 (35). The deliberate omission of the terms “common” and “respective” generated significant controversy, with developing countries uniformly criticizing the alteration as the product of intense pressure from developed states, which had publicly opposed the inclusion of standard CBDR-RC terminology without providing any transparent rationale for the terminological substitution (35).

This terminological shift carries profound legal implications. It undermines the established jurisprudential foundation of the CBDR-RC principle, eroding developing states’ ability to invoke decades of accumulated international legal precedent and doctrinal scholarship in future treaty interpretation (35). Specifically, replacing “common” with “shared” fundamentally reconfigures the principle’s normative architecture: whereas “common” connotes collective and indivisible responsibility in addressing global public goods challenges, “shared” implies individually apportioned and negotiable obligations (35). This semantic shift weakens the principle’s core equity rationale and dilutes developed states’ collective accountability for the historical and disproportionate benefits they have derived from the existing global health architecture (52). Furthermore, in the context of pathogen sharing, this reframing institutionalizes a hierarchical division of labor, positioning developing countries as providers of raw data and samples while reserving exclusive control over data analysis, intellectual property ownership, and commercial translation for developed states, without imposing corresponding binding capacity-building obligations (35). Moreover, the revised formulation risks enabling institutional overreach, whereby external actors can dictate the priorities of developing states’ capacity-building efforts rather than supporting endogenously determined pandemic response needs (35). Taken together, these shifts may weaken the principle’s core rationale: ensuring equitable burden-sharing and redress for structural inequities in global health governance (35).

The INB Bureau’s procedural justification did little to assuage these concerns. While the Bureau maintained that the Working Draft remained a preliminary, “living” document, its claim of broad editorial discretion carried substantive normative risks: as the Bureau explicitly acknowledged, it reserved the right to delete the SBDRC provision entirely, and the principle of CBDR-RC risked being omitted altogether from the subsequent Conceptual Zero Draft—the next critical milestone in the negotiation process (35).

#### 3.2.2 The zero draft (2023)

Building on discussions from INB2, the INB Bureau subsequently published the Conceptual Zero Draft (53) on 25 November 2022 for consideration at the 3rd INB meeting (INB3), marking the first formal inclusion of the CBDR-RC principle in the WHO Pandemic Agreement drafting process. This principle is explicitly integrated into the document in two key respects. The Draft’s preamble paragraphs 28, 30, 33 and 34 recognize developing countries’ PPPR burdens, capacity disparities, financial and technical support needs and the common but differentiated responsibility by all States Parties. Second, and more critically, the CBDR-RC principle was elevated to a binding guiding principle in Article 4.8 of the Conceptual Zero Draft, marking its culmination as a formal treaty norm.

Subsequently, on 1 February 2023, the INB Bureau released the official Zero Draft (54) to serve as the baseline text for formal negotiations at the 4th INB meeting (INB4). However, the Zero Draft eroded this normative advance, diluting the CBDR-RC principle through textual formulations that departed from its established interpretation, the insertion of discretionary qualifiers, and the structural neglect of equity provisions. These changes fundamentally departed from the established interpretation of the CBDR-RC principle in international law. The Zero Draft retained references to this principle in the preamble (paragraphs 36 and 49) and Article 4.8. However, three critical textual modifications undermined its legal force (55). First, Article 4.8 opened by framing health security as a universal state obligation (“All States are responsible for the health of their people”). This re-centering of the provision around a shared, undifferentiated duty signalled a marked retreat from the structural acknowledgement of asymmetry inherent in the CBDR-RC principle, which demands that common obligations be calibrated to national capacities. Second, the clause conditioned the bearing of differentiated responsibility on the possession of resources, specifying that “States that hold more resources relevant to pandemics, including pandemic-related products and manufacturing capacity”—rather than, for instance, those bearing greater historical responsibility for global health inequities or those with weaker health systems—“should bear … a commensurate degree of differentiated responsibility,” thereby narrowing its normative foundation to the mere possession of resources. Third, the insertion of the qualifying phrase “where appropriate” introduced broad state discretion into the determination of obligations, effectively rendering them voluntary rather than mandatory. This constituted a fundamental departure from the canonical interpretation of the CBDR-RC principle as established in international environmental law and as sought by developing countries in the pandemic context.

Beyond the textual dilution of Article 4.8, the Zero Draft structurally undermined equity through its treatment of key operational provisions (55). First, technology transfer (Article 7) and R&D obligations (Article 9) mixed binding language with hortatory qualifiers (“encourage,” “endeavour”), granting states broad implementation discretion. Second, supply chain provisions (Article 13) prioritized procedural mechanisms over enforceable equitable product distribution commitments, emphasizing health security surveillance over substantive equity. Most critically, financing clauses (Article 19) imposed no binding obligations on developed states, lacking quantified targets or CBDR-RC benchmarks.

In essence, the Zero Draft represented a substantial regression from the normative promise of the CBDR-RC principle enshrined in the Conceptual Zero Draft. While formally retaining this principle’s terminology, it appears to have weakened its legal force through non-binding language, voluntary mechanisms, and the strategic insertion of discretionary qualifiers. This weakening of the principle’s operational core created a critical gap between the WHO Pandemic Agreement’s proclaimed equity aspirations and its actual legal substance.

#### 3.2.3 The bureau’s text and revised bureau’s text (2023)

At the resumed fifth session of the INB in June 2023, the INB Bureau formally tabled its consolidated text, in which the status of the CBDR-RC principle emerged as one of the most contentious fault lines in the negotiations (56). Article 3.7 of the Bureau’s Text (54) presented three divergent options for this principle’s formulation, reflecting the fundamental impasse between developed and developing states. Option 7.A retained the full CBDR-RC formulation, acknowledging that “Governments have a responsibility for the health of their peoples” while recognizing that the unequal development of countries in health promotion and diseases control constitutes a shared global danger, and obligating resource-rich States to bear commensurate responsibilities. Option 7.B proposed a substantially weakened alternative under the heading “Common responsibilities and different capabilities”, diluting the normative force of the original CBDR-RC formulation by replacing codified international law terminology with vague capability-based language (56). Option 7.C proposed the outright deletion of the principle from the treaty. As developing countries had feared, this represented a significant retreat from both the Conceptual Zero Draft and Zero Draft, which had treated the principle as a settled normative foundation rather than a matter of contention (56). This proposal was met with strong opposition from developing countries, which condemned what they perceived as a fundamental erosion of equity commitments in favour of a framework that prioritized pathogen surveillance and sample sharing (57). The Proposal for the WHO Pandemic Agreement, tabled on 30 October 2023 (58), further weakened the principle of CBDR-RC, favoring a simpler formulation that merely acknowledged “different capacities” (59).

However, at INB-9 on 13 March 2024, the INB circulated to Member States the revised Bureau’s Text (60) and the principle of CBDR-CR is kept in the preamble and the Article 3.4. Specifically, Article 3.4 explicitly enshrined the CBDR-CR principle as one of the six foundational principles (60). Throughout this process, a deep and persistent North–South cleavage shaped competing textual submissions. Developed country delegations prioritized legally binding obligations on developing States to enhance pathogen surveillance and timely information sharing. Conversely, developing country blocs—led by the African Group, the Group of 77 and China, and the cross-regional Equity Group—insisted on reciprocal binding obligations for developed States to operationalize equity through three core mechanisms: (1) geographically diversified production of pandemic-related products; (2) mandatory technology transfer and intellectual property flexibilities; and (3) fair and equitable benefit-sharing arising from the utilization of pathogens and their genetic sequence data (15).

Against this backdrop, the INB Bureau assumed a highly unusual for a Member State-driven negotiating process role as an active mediator and drafting architect tasked with producing a consolidated text to reconcile deep divisions ahead of the 77th WHA in May 2024 (61). However, through opaque drafting practices, agenda control, and late-night informal sessions, the Bureau progressively diluted key equity commitments—such as converting mandatory access-and-benefit-sharing obligations into flexible targets and best-effort clauses (62). This procedural and substantive shift raised profound concerns among developing countries, particularly the Africa Group, regarding the prioritization of stringent surveillance and notification burdens over legally enforceable equity guarantees (15).

#### 3.2.4 The new draft negotiating text (2024)

There was a new proposal (63) for removing the CBDR-RC principle in the revised Bureau’s text on 23 April 2024. Subsequently, the new Draft Negotiating Text (64) introduced on 27 May 2024, representing a streamlined version based on the textual proposals made by Member States during the 9th session of INB. Notably, the Text fails to adequately embed the development divide between developed and developing countries in its obligation design, a flaw exacerbated by the absence of explicit recognition of the CBDR-RC principle in Article 3.

While the preamble acknowledges differences in national development levels and capacities, this framing is not translated into binding differentiated obligations across operational provisions. Most obligations under Articles 4 (Pandemic prevention and surveillance), 5 (One Health approach), 6 (Preparedness, readiness and health system resilience) and 7 (Health and care workforce) are framed in uniform language, qualified only by vague caveats such as “within the means and resources at its disposal” (Article 6.1, Article 6.2), “as appropriate”(Articles 4, 5, 6, 7) and “subject to the availability of resources” (Article 4.2, Article 5.2, Article 5.3), which fail to establish clear compliance benchmarks tailored to divergent national capacities. Furthermore, while Article 19 requires Parties to cooperate to strengthen pandemic capacities “particularly for developing country Parties”, it only establishes generic, non-binding cooperation obligations applicable to all Parties, and does not impose exclusive, enforceable assistance responsibilities on developed countries possessing superior financial and technological resources. This structural weakness is compounded by Article 20 on sustainable financing, which contains no specific quantified targets, mandatory contribution mechanisms, or differentiated financial obligations for developed countries. The proposed Coordinating Financial Mechanism (CFM) also lacks provisions guaranteeing dedicated, predictable funding streams for developing country implementation. While the Text contains multiple references to technical and financial assistance for developing countries, nearly all such provisions are heavily qualified with discretionary language, fail to identify specific responsible Parties or institutions, and postpone critical operational details to future COP decisions (65).

In conclusion, the text imposes binding surveillance obligations on developing countries while adopting non-binding “best endeavour” language on equity-related provisions, creating a regulatory imbalance that may limit the enforceable responsibilities of developed countries in building an equitable, solidarity-based pandemic prevention, preparedness and response regime.

#### 3.2.5 The pandemic agreement (2025)

Although the main text of the WHO Pandemic Agreement was formally adopted on 20 May 2025 as the world’s first dedicated international legally binding treaty framework on PPPR (66), the Agreement cannot yet proceed to signature under Article 31 because the opening for signature is linked to the adoption of the PABS Annex. The culmination of these three and a half years of fraught intergovernmental negotiations therefore remains incomplete without the settlement of the PABS System (1). Yet this final legal instrument erodes the normative substance of the CBDR-RC principle, resulting in a normative structure that nominally acknowledges development disparities and Global South equity demands, while weakening legally binding differentiated obligations from its substantive core.

At the level of core guiding norms, the Agreement’s Article 3 explicitly enshrines six foundational principles: the sovereign right of States; full respect for human dignity, human rights and fundamental freedoms; full respect for relevant international humanitarian law; equity as a goal, principle and outcome of pandemic action; solidarity in health emergencies; and the best available science and evidence as the basis for public health decision-making. Critically, the text pointedly excludes CBDR-RC as a standalone, binding guiding principle for the Pandemic Agreement’s interpretation and implementation. While the preamble acknowledges that cross-national differences in development levels engender divergent pandemic response capacities and calls for support from better-resourced nations, these preambular provisions hold only interpretive weight rather than binding force. In international law, treaty preambles merely outline contextual aspirations and cannot impose enforceable obligations like Article 3—the substantive core of the treaty that establishes its governing principles. Consequently, without translating these equity concerns into binding operative clauses, the Agreement’s core provisions fail to mandate concrete differentiated duties. Furthermore, while Article 3’s solidarity principle incorporates language recognizing “different levels of capacities and capabilities, particularly of developing countries”, this phrasing is purely descriptive and fails to translate the core of the CBDR-RC principle—differentiated legal obligations corresponding to national capabilities, and the lead responsibility of developed states to provide enabling support—into normative guidance for the Agreement’s operationalization.

In the design of substantive obligations, the Agreement adopts a *de facto* “one-size-fits-all” model that is difficult to reconcile with the CBDR-RC framework. Article 4 on pandemic prevention and surveillance imposes uniform capacity-building and implementation obligations on all Parties, qualified only by the vague caveats of “taking into account national capacities and national and regional circumstances” and “subject to the availability of resources”. It neither establishes tiered or differentiated legal obligations matched to the economic and technological capabilities of developed and developing countries, nor creates corresponding duties for developed states to offset the implementation costs for lower-capacity nations. Regarding enabling implementation support, Article 17 on international cooperation and support for implementation, and Article 11 on technology transfer and related know-how, are overwhelmingly framed as non-binding “best endeavour” clauses. Technology transfer provisions are explicitly predicated on terms “as mutually agreed” between parties, while international cooperation measures are qualified by “subject to national and/or domestic law and available resources”. Neither article establishes legally binding, enforceable obligations on developed countries to provide predictable technical and financial support to developing country Parties. Most critically, the Agreement’s sustainable financing regime under Article 18 fails to operationalize the CBDR-RC principle’s core requirement of differentiated financial responsibility. The established CFM directly repurposes the existing framework under the amended IHR, rather than creating an independent, dedicated financing mechanism with mandatory, assessed funding obligations for developed countries. While the Agreement allows for the exploration of innovative financing sources, it imposes no binding financial commitments on developed states, eliminating the core accountability mechanism of the CBDR-RC principle in global pandemic governance.

Ultimately, the Agreement only incorporates fragmented, descriptive elements of the CBDR-RC principle in its preamble and the solidarity clause of Article 3, while rejecting its institutionalization as a core guiding norm and translating none of its requirements into binding differentiated obligations. This structural design of “uniform mandatory obligations, soft-law enabling support” fails to redress the structural inequities in global health governance laid bare by the COVID-19 pandemic. With the exception of the binding legal framework establishing the PABS System (whose operational details remain to be finalized in a subsequent annex instrument), the Agreement relies heavily on *ad hoc* voluntary commitments in all critical equity-related domains, significantly undermining its stated core objective of advancing equity in global pandemic prevention, preparedness and response.

In sum, while the final adopted text of the WHO Pandemic Agreement in May 2025 recognized equity as a guiding norm, CBDR-RC was largely confined to the preambular context and was not incorporated into operative provisions establishing binding obligations. Consequently, the ongoing negotiations concerning the PABS Annex represent the remaining regulatory opportunity to translate CBDR-RC from a normative principle into concrete mechanisms, including benefit-sharing arrangements, capacity-calibrated obligations, and trigger-based financing commitments. Table 2 visually summarizes the chronological evolution of CBDR-RC throughout the WHO Pandemic Agreement negotiations, highlighting the progressive transformation, qualification, and eventual narrowing of CBDR-RC language from early negotiating drafts to the adopted treaty text and the continuing PABS Annex process.

**Table 2:** Evolution of the CBDR-RC principle in WHO Pandemic Agreement negotiations (December 2021–July 2026).

Date/timeframe	Negotiation milestone and document draft	Textual trajectory and status of CBDR-RC principle
December 2021	WHA Special Session	Establishment of the INB → Mandate to develop a legally binding instrument on PPPR
July 2022	Working Draft	CBDR-RC terminology replaced by SBDRC → Initial uncertainty regarding the legal recognition of differentiation
November 2022–February 2023	Conceptual Zero Draft and Zero Draft	CBDR-RC formally reintroduced as a guiding principle → Included within equity-related provisions but qualified by discretionary language (“where appropriate”, “as applicable”)
June–October 2023	Bureau’s Texts and State Proposals	Intensified North–South divergence Competing approaches emerged: (1) retaining explicit CBDR-RC language; (2) replacing CBDR-RC with capacity-based terminology; (3) removing CBDR-RC from treaty text
March–May 2024	Revised Bureau’s Text and New Draft Negotiating Text	CBDR-RC removed from operative general principles → Greater emphasis placed on uniform surveillance, notification, and preparedness obligations
May 2025	Adoption of WHO Pandemic Agreement (78th WHA)	Main treaty text officially adopted CBDR-RC not included as an operative binding obligation → Equity retained as a guiding principle, including through Article 3.4
2025–July 2026	PABS Annex Negotiations (ongoing)	Remaining institutional window to operationalize CBDR-RC through: • Binding benefit-sharing mechanisms • Differentiated capacity-building obligations • Technology transfer arrangements • Trigger-based financing mechanisms

This timeline illustrates the textual and institutional trajectory of the CBDR-RC principle during the negotiation of the WHO Pandemic Agreement. It demonstrates that CBDR-RC moved from uncertain recognition in early negotiating drafts, to partial inclusion as a guiding principle, and ultimately to limited incorporation outside the Agreement’s binding provisions. The ongoing PABS Annex negotiations represent the remaining opportunity to translate differentiated responsibility into operational mechanisms for equitable pandemic governance.

## 4 Prospects to incorporate the CBDR-RC principle in the PABS annex

Existing international cooperation provisions in international health law, especially including those in the WHO Pandemic Agreement and the revised IHR, outline broad commitments to technology transfer, financial assistance, and capacity-building for developing countries, yet lack concrete, detailed legal obligations, the core of the CBDR-RC principle. The WHO Pandemic Agreement fails to incorporate the CBDR-RC principle, raising questions regarding the implementation of equity in global health governance. While the principles of equity and solidarity are widely endorsed in international law, its practical effect may remain limited without enforceable, substantive legal mechanisms to translate it into practice. Although the inclusion of the CBDR-RC principle was proposed by some States as an amendment to the IHR, this principle was not adopted in the amended IHR (67). The Review Committee pointed out that the IHR do not explicitly provide for differentiated responsibilities among States Parties, but rather requires cooperation among them all, suggesting that it may not be the most appropriate instrument for introducing this principle (68).

### 4.1 Justification of incorporation of the CBDR-RC principle in the PABS annex

The absence of robust CBDR-RC norms risks perpetuating pre-existing inequities in global health governance (69). The principle of CBDR-RC ought not to be excluded from international health law. On the contrary, it can be normatively reframed and calibrated to align with the established doctrinal framework of this legal regime. One of the major shortcomings of the IHR 2005 is that it fails to effectively recognize the development divide, treating unequal States equally. Only through the incorporation of the CBDR-RC principle for a comprehensive system of PPPR, it would be possible to address inequalities between the Global North and the Global South, thus changing the narrative on fairness and equity in international health law (9).

Incorporating CBDR-RC into international health law delivers three core normative and institutional benefits. First, it translates the ambiguous principles of equity and solidarity into a justiciable, operational principle that can be codified in binding legal instruments such as the WHO Pandemic Agreement and the revised IHR. Second, it converts non-binding political commitments into enforceable legal obligations with clear implementation and oversight mechanisms, thereby strengthening the accountability framework. Finally, it provides a normative foundation to establish standardized legal procedures for defining, allocating, and evaluating differentiated state responsibilities, serving as a critical first step toward rebuilding a more equitable and effective international health regime. In this regard, the COVAX Facility provides a useful example of international health cooperation grounded in the CBDR-RC principle. Developed States would have borne a duty to cooperate adequately and fairly in the initiative, and vaccine distribution would have been based on a generalized targeted allocation scheme (70).

### 4.2 Operationalizing the CBDR-RC principle in the PABS annex: tiered obligations and institutional safeguards

Following the adoption of the WHO Pandemic Agreement, global attention has shifted decisively to the PABS Annex (71), upon which the Agreement’s legitimacy and effectiveness will largely hinge (72). The PABS negotiations have continued to evolve. At its seventh meeting, held from 6 to 17 July 2026, the IGWG further streamlined the draft PABS Annex while continuing consultations on unresolved issues, including the contractual arrangements underpinning the system, the structure of laboratory networks for sharing and handling pathogens and their sequence information, and the definition of benefits arising from pathogen sharing. WHO reported on 20 July 2026 that further negotiations would be required to finalize the Annex. The Annex therefore remains under negotiation, making the distribution of responsibilities and benefits within the emerging PABS architecture an open question of institutional design.

The PABS Annex (73)—the draft annex contemplated by Article 12 of the WHO Pandemic Agreement (74)—proposes a two-pillar architecture: States Parties’ obligation to share pathogen samples and sequence data with WHO-coordinated networks, and the equitable sharing of resulting benefits, including vaccines, diagnostics, and therapeutics, based on public health risk and need (75). This article reaffirms states’ sovereign rights over their biological resources in the PABS system, yet underscores that timely access to pathogen samples and sequence information remains indispensable for R&D (72). The continuation of negotiations beyond the 79th WHA also reflects persistent disagreement over how equity should be translated into concrete obligations and benefit-sharing arrangements within the PABS system (76). Against this background, the operationalization of the CBDR-RC principle within the PABS Annex becomes particularly important for addressing structural inequities in global health governance (72). Proposals by developing countries for its inclusion deserve serious consideration, as they align with the broader imperatives of equity and fairness in international law and could mitigate the disproportionate health and socio-economic impacts of future pandemics on vulnerable nations.

Yet, as currently drafted, the PABS Annex fails to embed equity in a legally meaningful form. While the draft PABS Annex formally enshrines the “equal footing” between pathogen sharing and benefit-sharing (77), it frames equity exclusively through vague, aspirational flexibility clauses and fails to codify binding differentiated obligations calibrated to States’ historical responsibilities and current capacities. Specifically, it does not codify binding, differentiated obligations calibrated to the historical responsibilities and current capabilities of developed and developing States (52). This omission is not merely a technical drafting gap; it represents a significant normative deficit that risks reproducing the structural inequities witnessed during COVID-19, where high-income countries secured disproportionate vaccine coverage while resource-contributing low-income countries were largely excluded from the benefits of their shared biological materials.

The legal analysis above provides the normative basis for addressing this gap, but it does not itself establish the specific institutional mechanisms proposed below. It establishes the relevance of CBDR-RC, equity, differentiated capacities, and benefit-sharing to pandemic preparedness and PABS, while identifying the absence of a sufficiently differentiated allocation of responsibilities within the emerging PABS framework. Building on these legal and normative findings, this article therefore proposes three institutional mechanisms—tiered obligations, technology transfer, and trigger-based financing—to operationalize CBDR-RC within the PABS framework. These mechanisms are advanced as the author’s proposed model for future institutional design, rather than as obligations already established under the Pandemic Agreement or the PABS Annex.

To close this gap, the CBDR-RC principle must be elevated from a background normative aspiration to a core organizing principle of the PABS system. This article therefore proposes integrating the CBDR-RC principle into the operational architecture of the PABS Annex through three mutually reinforcing mechanisms: (1) tiered obligations calibrated to national capacity levels, moving beyond a rigid developed/developing binary; (2) conditional technology transfer provisions that link access to pathogen data with enforceable commitments to share manufacturing know-how; and (3) trigger-based financing arrangements that activate pre-negotiated funding obligations during public health emergencies of international concern. Although these mechanisms primarily differentiate responsibilities among States, their implementation would necessarily involve private manufacturers that possess substantial technological, manufacturing, and commercial capacities. States would therefore translate their differentiated international responsibilities into regulatory, contractual, procurement, and financial arrangements governing or incentivizing private-sector participation. These mechanisms collectively provide a structured, legally coherent pathway to render the “equal footing” commitment a substantive reality rather than a rhetorical aspiration.

The legal and empirical justification for such differentiation is supported by both legal and empirical considerations. As South Africa emphasized on behalf of the Africa Group, the African continent has repeatedly shared its pathogen resources without receiving equitable returns, thereby framing the PABS System as a critical corrective to these longstanding inequities (71). Countries most likely to contribute pathogen materials are biodiverse equatorial nations—such as Brazil, the Democratic Republic of the Congo, and Indonesia—which generate abundant genomic sequence data due to high rates of human-animal interaction. Conversely, those best positioned to capture the resulting benefits are countries with advanced technological, financial, and industrial infrastructure, including the United States, the European Union member states, and Japan (52). This asymmetric dynamic mirrors structural inequities documented in prior access-and-benefit-sharing regimes, such as the ITPGRFA (78) and the Pandemic Influenza Preparedness (PIP) Framework (79). Consequently, over decades, developed nations have consolidated substantial technological advantages, biomanufacturing capacities, and patent monopolies through their dominance of global pathogen surveillance networks and biomedical R&D infrastructure, while resource-limited states have borne disproportionate pandemic burdens. This historical and structural inequity calls for a legal response under the CBDR-RC principle, providing a basis for preferential allocation of benefits, tiered compliance timelines, and dedicated capacity-building support for developing countries under the PABS framework (51).

Operationally, the CBDR-RC principle should be institutionalized through tiered commitments across the entire pathogen surveillance–R&D–manufacturing–distribution value chain, consistent with the draft PABS Annex’s existing flexibility provisions (80). Building on Article II.A.3 of the draft PABS Annex, it is proposed that extended transitional compliance periods (3–5 years) be granted to developing countries with limited laboratory and regulatory infrastructure to align their national ABS frameworks with the PABS System and meet standardized pathogen sharing and reporting requirements. Conversely, developed country Parties, as the primary jurisdiction of incorporation for most Participating Manufacturers, must assume enhanced regulatory obligations to ensure that these commercial entities comply with binding benefit-sharing commitments. Such State responsibilities could be implemented, where appropriate, through domestic regulation, public procurement conditions, licensing arrangements, or contractual requirements attached to public financing and market access. In this way, private manufacturers would participate in implementing differentiated State responsibilities without becoming direct bearers of the CBDR-RC principle itself. To operationalize equity in the Draft Annex, model PABS Contracts should explicitly mandate that a binding minimum percentage of real-time production of vaccines, diagnostics, and therapeutics be allocated to the WHO-coordinated distribution mechanism for equitable, needs-based deployment during public health emergencies—thereby transforming the current aspirational language into enforceable provisions.

Furthermore, consistent with the CBDR-RC principle’s call for intra-group differentiation, developing countries must be further stratified to reflect their heterogeneous capacities and vulnerabilities, moving beyond the outdated binary framework. This refined stratification aligns with the WHO Secretariat’s mandate to prioritize resources based on public health risk and unmet need. Specifically, rather than treating the Global South as a monolith, the Pandemic Agreement should adopt a nuanced stratification based on structural vulnerability. Developing countries that lack domestic pharmaceutical production capacity face distinct systemic barriers: Landlocked Developing Countries (LLDCs) suffer from exorbitant supply chain and transit costs during border closures; Small Island Developing States (SIDS) experience severe geographic isolation and logistical delays; and LDCs grapple with limited economic scale and baseline health integration. Consequently, these groups—LDCs, LLDCs, and SIDS—should be eligible for targeted and prioritized support measures. These include: (1) first-priority access to WHO-allocated vaccines, therapeutics, and diagnostics during public health emergencies; (2) dedicated technical and financial assistance to establish and participate in WHO-coordinated laboratory networks (81); and (3) preferential eligibility for all technology transfer and capacity-building programs administered by the WHO Secretariat. The proposed differentiation would also operate across the State–manufacturer interface. While capacity and vulnerability would primarily inform the responsibilities and support available to States, the implementation of technology transfer and equitable production would require States to engage manufacturers through licensing, research partnerships, procurement arrangements, and other appropriate contractual mechanisms. Private manufacturers could thereby contribute to the production and distribution of pandemic countermeasures while States retain responsibility for translating CBDR-RC into applicable regulatory and institutional arrangements. This approach would allow the PABS framework to address the practical reality that technological and manufacturing capabilities are often concentrated in private firms, while avoiding the extension of CBDR-RC as a direct international legal obligation to non-State actors.

Finally, the institutional architecture for implementing CBDR-RC must be strengthened. The Conference of the Parties (COP), serving as the supreme governing and oversight body of the PABS System (82), should be explicitly tasked with integrating historical responsibility and national capacity metrics into the calculation of monetary contributions from Participating Manufacturers (83) and the standard terms of PABS Contracts. Specifically, mandatory annual contributions should be calibrated based on two complementary factors: (1) the commercial benefits derived by manufacturers from access to PABS Materials and Sequence Information; and (2) the historical and current capacity of the state in whose jurisdiction the manufacturer is incorporated to contribute to global pandemic preparedness. Such contributions could be structured through the relevant State and PABS institutional arrangements so that manufacturers’ financial obligations are connected to the commercial benefits they derive from the system while remaining embedded within a State-mediated framework. Furthermore, the PABS Advisory Group, which provides scientific and technical advice (84), should be formally mandated to deliver evidence-based recommendations on differentiated compliance, equitable benefit allocation, and the effectiveness of capacity-building support. Crucially, the Advisory Group must include guaranteed proportional representation from developing country experts, with at least 50 % of its members drawn from LDCs, LLDCs, and SIDS.

Ultimately, the credibility and effectiveness of the PABS System will depend on a robust compliance mechanism. The current draft PABS Annex merely provides for a facilitative compliance procedure (73), which lacks the authority to address systematic or material breaches of PABS obligations (52). Establishing a mechanism endowed with the competence to consider cases of non-compliance—particularly those concerning the failure of Participating Manufacturers to meet their benefit-sharing commitments—and to apply appropriate, proportionate measures is indispensable. Where manufacturers participate through contractual or regulatory arrangements established by States, non-compliance could also be addressed through proportionate contractual, regulatory, procurement, or financial consequences under the applicable domestic and PABS frameworks. Such a mechanism would not only ensure that tiered, differentiated obligations are rendered enforceable, but would also strengthen the WHO’s institutional legitimacy and set a transformative precedent for accountability across the broader landscape of global health governance, enhancing the WHO’s authority to enforce the amended IHR and other public health emergency frameworks (52).

## 5 Discussion

The adoption of the WHO Pandemic Agreement in May 2025 marked a historic milestone in global health law. However, the limited incorporation of the CBDR-RC principle into the operative provisions of the Agreement leaves unresolved questions concerning how equity should be translated into enforceable institutional mechanisms. As global health governance transitions toward negotiating the details of the PABS Annex, this section interprets the findings of this study in light of existing scholarship, identifies the theoretical and practical implications of the analysis, acknowledges methodological and institutional limitations, and develops policy and regulatory implications for strengthening future pandemic governance.

Three analytical findings emerge from this study. First, CBDR-RC has evolved beyond a sector-specific environmental principle and represents a broader governance logic for addressing structural inequalities in global collective action. Second, comparative experiences from ABS regimes demonstrate that equity principles require institutional mechanisms and enforceable obligations rather than merely rhetorical recognition. Third, the negotiation trajectory of the WHO Pandemic Agreement reveals that the effectiveness of CBDR-RC depends not only on its formal legal recognition but also on the design of implementation mechanisms capable of aligning obligations with differentiated capacities.

### 5.1 Situating findings in prior research: beyond environmental law and traditional ABS frameworks

Previous scholarship has predominantly examined CBDR-RC within the context of international environmental law, emphasizing its relationship with historical responsibility, distributive justice, and differentiated treaty obligations (85). While this literature has provided important theoretical foundations, it has generally focused on environmental governance and has paid limited attention to whether CBDR-RC can provide a legal framework for addressing structural inequalities in global health emergencies.

The findings of this study extend this literature in two respects. First, they demonstrate that the underlying rationale of CBDR-RC—namely, that common global challenges may require differentiated obligations according to capacity, contribution, and vulnerability—is not inherently confined to environmental regulation. Instead, comparative analysis across climate governance, trade law, and global health instruments indicates that differentiated responsibility has become a recurring institutional technique within international law. Building on this finding, this study proposes that differentiation in pandemic governance should be based on identifiable and periodically reviewable indicators rather than on a fixed developed/developing-country classification. Capacity could be assessed by reference to fiscal and financing capacity, health-system and laboratory capacity, technological and manufacturing capability, and access to relevant research and production infrastructure. Vulnerability could be assessed by considering disease burden, health-system fragility, exposure to cross-border health risks, and the socioeconomic consequences of pandemics. Contribution could encompass States’ demonstrated and ongoing contributions to pandemic preparedness and response, including pathogen surveillance, pathogen and sequence-data sharing, research and development, financing, and the provision of relevant biological or scientific resources. These indicators are proposed as an evidentiary basis for differentiation, rather than as mechanically determinative thresholds. In functional terms, capacity would primarily inform the extent of implementation responsibilities, vulnerability the degree of flexibility and support, and contribution the allocation of responsibilities and equitable access to benefits. Vulnerability should not operate as a blanket exemption from participation in the PABS system. Rather, it should primarily affect the timing, scope, and means of implementation, including access to financing, technology transfer, technical assistance, and extended compliance periods. This distinction preserves the universal character of the PABS framework while allowing implementation responsibilities to reflect materially different national circumstances. Second, this study challenges the assumption that applying CBDR-RC to global health would necessarily reproduce a rigid North–South allocation model. The evolution of differentiation mechanisms, particularly under the Paris Agreement and health-related treaty practice, suggests a shift toward flexible, capacity-calibrated obligations rather than fixed categorical divisions between developed and developing States. By drawing on the 2025 ICJ Advisory Opinion as jurisprudential support for the equity-oriented and capacity-sensitive dimensions of CBDR-RC, this study contributes to global health law scholarship by providing a stronger legal and normative basis for examining the potential adaptation of differentiated responsibilities to pandemic governance. Furthermore, this study builds upon existing critiques of ABS frameworks, including the PIP Framework and the ITPGRFA. Traditional ABS regimes have revealed persistent structural asymmetries: developing countries often provide biological resources, pathogen samples, or genomic information, while technological and manufacturing advantages remain concentrated among high-income States and private pharmaceutical actors.

Against this background, the central contribution of this study is not to propose a new form of resource exchange, but to reconceptualize ABS as an institutional mechanism for distributive justice. The proposed operationalization framework—comprising differentiated compliance pathways, conditional technology-transfer mechanisms, and capacity-based financing arrangements—seeks to move beyond voluntary solidarity models by embedding equity considerations into legally structured governance arrangements.

### 5.2 Study limitations and real-world implementation challenges

While this study provides a legal and institutional framework for operationalizing CBDR-RC within the PABS Annex, several limitations and implementation challenges should be acknowledged. First, the analysis necessarily reflects the perspective and constraints of doctrinal legal research. Although legal analysis can identify normative possibilities, institutional inconsistencies, and treaty design options, it cannot fully capture the political bargaining dynamics, economic interests, and geopolitical considerations that ultimately determine treaty outcomes. Therefore, the proposed framework should be understood as an institutional design analysis rather than a prediction that political consensus on CBDR-RC will emerge automatically. Despite the legal rationale supporting capacity-sensitive obligations, opposition from some developed States remains significant. Concerns regarding potential financial liability, intellectual property implications, and mandatory technology-transfer requirements continue to shape negotiations. Consequently, the future operationalization of CBDR-RC within the PABS Annex will depend not only on legal arguments but also on diplomatic negotiation, coalition-building, and political compromise.

Second, the proposed framework faces significant implementation challenges arising from the relationship between States and private pharmaceutical actors. Treaty obligations alone may not guarantee compliance by multinational enterprises unless accompanied by effective domestic regulatory mechanisms, procurement conditions, and contractual arrangements. In particular, the effectiveness of benefit-sharing and technology-transfer obligations may depend on whether States are willing and able to incorporate international commitments into domestic regulatory frameworks governing public funding, licensing, and pharmaceutical production.

Third, the regulatory environment remains dynamic. Because negotiations concerning the PABS Annex continue to evolve, the legal formulations examined in this study represent a specific stage of institutional development rather than a final regulatory outcome. Future negotiations may modify the scope, language, and enforceability of equity-related obligations. Accordingly, the conclusions of this study should be understood as identifying legally feasible pathways rather than providing a definitive assessment of the final treaty architecture.

### 5.3 Concrete policy and regulatory implications

The findings of this study suggest several policy and regulatory implications for strengthening future pandemic governance. Rather than treating CBDR-RC as a general statement of solidarity, policymakers should focus on translating differentiated responsibility into operational mechanisms capable of producing measurable institutional outcomes.

#### 5.3.1 Institutional design of the WHO Pandemic Agreement and PABS annex

At the international level, the WHO and relevant governing bodies should consider developing standardized PABS implementation mechanisms that incorporate differentiated obligations according to manufacturing capacity, technological capability, and public-health vulnerability. Such mechanisms may include model contractual arrangements, transparent benefit-sharing criteria, and predictable financing structures to ensure that access to pathogen resources is accompanied by equitable access to resulting medical countermeasures. Rather than imposing a uniform obligation on all participating actors, the regulatory framework should adopt a capacity-sensitive approach in which obligations are calibrated according to the resources, technological capabilities, and institutional capacities of relevant stakeholders.

#### 5.3.2 Domestic regulatory reforms in high-income states

High-income States should consider integrating global equity requirements into domestic regulatory frameworks governing publicly funded pandemic research, procurement programs, and pharmaceutical innovation. Public financing mechanisms and advance procurement agreements could incorporate contractual conditions requiring beneficiaries to participate in international benefit-sharing arrangements and support equitable access during public health emergencies. In addition, domestic legal frameworks may need to provide greater flexibility for technology transfer, licensing arrangements, and manufacturing cooperation during emergencies, while maintaining an appropriate balance between innovation incentives and global health equity.

#### 5.3.3 Capacity-building strategies for developing countries

For developing countries, especially those with limited manufacturing and surveillance capacity, effective participation in CBDR-RC-based governance requires investment in domestic institutional capacity. This includes strengthening genomic surveillance systems, laboratory infrastructure, regulatory institutions, and regional manufacturing capabilities. Regional cooperation mechanisms, including pooled procurement arrangements and collaborative manufacturing platforms, may also enhance bargaining capacity and reduce structural dependence on external suppliers during future pandemics.

Overall, this study argues that the future effectiveness of global pandemic governance will depend not merely on recognizing equity as a normative aspiration, but on designing legal institutions capable of translating differentiated responsibility into operational obligations. CBDR-RC therefore should not be understood as a redistributional exception to international health law, but rather as a governance principle that enables more sustainable and resilient collective action in addressing global health emergencies.

## 6 Conclusion

The transboundary transmissibility and catastrophic socioeconomic impacts of pandemics constitute an existential global public health threat, with no nation immune to their consequences. The COVID-19 pandemic exposed profound structural inequities in global health governance, underscoring the urgency of multilateral cooperation. Rather than merely reflecting a settled collective resolve, the WHO Pandemic Agreement and its PABS Annex represent a critical opportunity to genuinely operationalize the CBDR-RC principle within the global PPPR legal architecture, testing whether the equity and solidarity principles enshrined in the WHO Constitution and the revised IHR can be translated into binding commitments.

The principle of CBDR-RC has evolved into an important normative and institutional approach to addressing asymmetries in historical responsibilities, capacities and pandemic burdens. Applying this approach in the PABS context requires a nuanced, context-specific approach across the entire pathogen-related surveillance–R&D–manufacturing–distribution continuum, including targeted preferential treatment for developing countries, sub-categorization of developing nations based on vulnerability and capacity, and a dedicated implementation mechanism covering financial support, technology transfer and capacity-building.

Incorporating CBDR-RC considerations into the binding provisions of the PABS Annex is legally and pragmatically justified. It would help address COVID-19-era inequities, secure broad consensus on the final agreement, and ensure its implementation delivers tangible benefits to the most vulnerable.

## Data Availability

The original contributions presented in the study are included in the article/supplementary material, further inquiries can be directed to the corresponding author.

## References

[1] Huang Y Jiang S Kumah E. China and the WHO pandemic treaty: a dive into stance, underpinnings, and implications. Front Public Health. (2024) 12:1335751. doi: 10.3389/fpubh.2024.1335751 PMC10865500 38356946

[2] United Nations. Who chief declares end to covid-19 as a global health emergency (2023). Available online at: https://news.un.org/en/story/2023/05/1136367 (Accessed May 6, 2026).

[3] Borges LC de Menezes HZ Crosbie E. From aspiration to action: aligning the pandemic agreement with equity in vaccine access; a response to recent commentaries. Int J Health Policy Manag. (2025) 14:9192. doi: 10.34172/ijhpm.9192, 40767241 PMC12337161

[4] Hampton A-R Eccleston-Turner M Rourke M Switzer S. Equity’ in the pandemic treaty: the false hope of ‘access and benefit-sharing. Int Comp Law Q. (2023) 72:909–43. doi: 10.1017/S0020589323000350 PMC10209987 37226758

[5] United Nations Department of Economic and Social Affairs. Covid-19 vaccination in LDCs—Status Report of 30 September 2022 (2022). Available online at: https://policy.desa.un.org/sites/default/files/inline-images/eapd2023/LDC-vaccine-30-Sep-22.pdf (Accessed May 6, 2025).

[6] Chen W. Study on major legal issues and solutions in pandemic treaty negotiations. Front Public Health. (2024) 12:1413036. doi: 10.3389/fpubh.2024.1413036, 39371209 PMC11449756

[7] Wang C Zhang Y. Common but differentiated responsibilities and respective capabilities as a guiding principle in international health law in times of pandemics. Neth Yearb Int Law. (2020) 51:275. doi: 10.1007/978-94-6265-527-0_9

[8] Kuznetsova L. COVID-19: the world community expects the World Health Organization to play a stronger leadership and coordination role in pandemics control. Front Public Health. (2020) 8:470. doi: 10.3389/fpubh.2020.00470, 33014970 PMC7505920

[9] Di Lullo L. Striving for fairness: a critical examination of the ‘common but differentiated responsibilities and capabilities’ principle in international health law. Int Community Law Rev. (2024) 26:228. doi: 10.1163/18719732-bja10121

[10] World Health Organization. World health assembly adopts historic pandemic agreement to make the world more equitable and safer from future pandemics (2025). Available online at: https://www.who.int/news/item/20-05-2025-world-health-assembly-adopts-historic-pandemic-agreement-to-make-the-world-more-equitable-and-safer-from-future-pandemics (Accessed May 6, 2026).

[11] World Health Organization. Amended International Health Regulations enter into force (2025). Available online at: https://www.who.int/news/item/19-09-2025-amended-international-health-regulations-enter-into-force#:~:text=Today%20marks%20a%20milestone%20in%20global%20health%20governance,shaped%20by%20hard-earned%20lessons%20of%20the%20COVID-19%20pandemic (Accessed May 6, 2026).

[12] World Health Organization. Countries make progress on WHO Pandemic Agreement annex on pathogen access and benefit sharing system (2025). Available online at: https://www.who.int/news/item/07-11-2025-countries-make-progress-on-who-pandemic-agreement-annex-on-pathogen-access-and-benefit-sharing-system (Accessed May 6, 2026).

[13] Sirohi Nishant. Is there a case for “common but differentiated responsibility” in global health? Geneva Health Files (2023). Available online at: https://genevahealthfiles.com/wp-content/uploads/2024/01/FEATURED-STORY-5-CBDR-IN-GLOBAL-HEALTH_compressed.pdf (Accessed May 6, 2026).

[14] Rajamani L. Differential Treatment in International Environmental Law. Oxford: Oxford University Press (2006). p. 133.

[15] Third World Network. World Health Organization: member states to hold another pandemic instrument negotiations round amidst wide divergence (2024). Available online at: https://www.twn.my/title2/health.info/2024/hi240401.htm (Accessed May 6, 2026).

[16] Wang S. Revisiting the common but differentiated responsibility principle in the prevention and control of marine plastic pollution. Int J Mar Coast Law. (2025) 40:423–43. doi: 10.1163/15718085-bja10231

[17] International Court of Justice. Obligations of states in respect of climate change, Advisory Opinion (2025), paras. 73, 78, 301–308, 364–365, 379.

[18] Erondu NA Sharma VD Mulumba M. Decolonial framings in global health law: redressing colonial legacies for a just and equitable future. Journal of Law, Medicine & Ethics. (2025) 53:76–8. doi: 10.1017/jme.2025.25, 40223658

[19] World Health Organization Commission on Social Determinants of Health. Closing the gap in a generation: health equity through action on the social determinants of health (2008). Available online at: https://iris.who.int/server/api/core/bitstreams/cb08095c-55c8-484e-bff6-0e9c78fd38dd/content (Accessed May 6, 2026).

[20] United Nations. Declaration of the United Nations Conference on Environment and Development (1992). 31 ILM 874 (Rio Declaration) principle 7.

[21] International Court of Justice. Obligations of states in respect of climate change, Advisory Opinion (2025), para. 148.

[22] International Court of Justice. Obligations of states in respect of climate change, Advisory Opinion (2025), paras. 148–151.

[23] Roeben V Amakoromo M. Responsibility, solidarity and their connections in international law: towards a coherent framework. Neth Yearb Int Law. (2020) 51:34. doi: 10.1007/978-94-6265-527-0_2

[24] Verma A. Adapting common but differentiated responsibility to the global cooperation for COVID-19 response (2020). Available online at: https://jia.sipa.columbia.edu/news/adapting-common-differentiated-responsibility-global-cooperation-covid-19-response (Accessed May 6, 2026).

[25] Oppenheim L. International Law: A Treatise. Vol. 1 Peace. London: Longmans, Green and Co. (1912). p. 20 “But as members of the community of nations they are equals, whatever differences between them may otherwise exist. This is a consequence of their sovereignty and of the fact that the Law of Nations is a law between, not above, the States”.

[26] Rajamani L. Differential Treatment in International Environmental Law. Oxford: Oxford University Press (2006). p. 158–72.

[27] Cullet P. Differential Treatment in International Environmental Law. London: Routledge (2003). p. 21–31.

[28] International Court of Justice. Obligations of states in respect of climate change, Advisory Opinion (2025), para. 151.

[29] Sands P Peel J Fabra A MacKenzie R. Principles of International Environmental Law. London: Cambridge University Press (2018). p. 244.

[30] World Health Organization. Declaration of Alma-Ata, International Conference on Primary Health Care (1978), para II.

[31] United Nations Committee on Economic, Social and Cultural Rights. CESCR general comment No. 14: the right to the highest attainable standard of health (Article 12 of the International Covenant on Economic, Social and Cultural Rights) (2000), E/C.12/2000/4, para. 40.

[32] Boyle A Redgwell C. Birnie, Boyle & Redgwell’s International Law and the Environment. London: Oxford University Press (2021). p. 149.

[33] World Health Organization. Intergovernmental negotiating body sessions. Available online at: https://apps.who.int/gb/inb/index.html (Accessed May 6, 2026).

[34] World Health Organization. Working draft, presented on the basis of progress achieved, for the consideration of the Intergovernmental Negotiating Body at its second meeting (2022). Available online at: https://apps.who.int/gb/inb/pdf_files/inb2/A_INB2_3-en.pdf (Accessed May 9, 2026).

[35] Ramakrishnan Nithin WHO: developed countries oppose CBDR inclusion in new pandemic instrument (2022). Available online at: https://www.twn.my/title2/health.info/2022/hi220702.htm (Accessed May 9, 2026).

[36] Article 4 (9) Shared but Differentiated Responsibilities and Capabilities—Full Consideration and Prioritization are Required of the specific Needs and special Circumstances of Developing Country Parties, Especially those that (i) are Particularly Vulnerable to Adverse Effects of Pandemics; (ii) do not have Adequate Conditions to Respond to Pandemics; and (iii) Would have to bear a Disproportionate or abnormal Burden.

[37] World Health Organization. Third meeting of the intergovernmental negotiating body, South Africa statement (2022). Available online at: https://apps.who.int/gb/inb/e/e_inb-3.html (Accessed May 9, 2026).

[38] World Health Organization. Third meeting of the intergovernmental negotiating body, Brazil statement (2022). Available online at: https://apps.who.int/gb/inb/e/e_inb-3.html (Accessed May 9, 2026).

[39] World Health Organization. Third meeting of the intergovernmental negotiating body, China statement (2022). Available online at: https://apps.who.int/gb/inb/e/e_inb-3.html (Accessed May 9, 2026).

[40] The Group for Equity is composed of the following countries: 1. Argentina, 2. Bangladesh, 3. Botswana, 4. Brazil, 5. China, 6. Colombia, 7. Dominican Republic, 8. Egypt, 9. El Salvador, 10. Eswatini, 11. Ethiopia, 12. Fiji, 13. Guatemala, 14. India, 15. Indonesia, 16. Iran, 17. Kenya, 18. Malaysia, 19. Mexico, 20. Namibia, 21. Pakistan, 22. Palestine, 23. Paraguay, 24. Peru, 25. The Philippines, 26. South Africa, 27. Tanzania, 28. Thailand, 29. Uruguay.

[41] Third World Network. TWN info service on biodiversity and traditional knowledge (2023). Available online at: https://www.twn.my/title2/biotk/2023/btk231103.htm (Accessed May 9, 2026).

[42] Third World Network. WHO:INB bureau to publish revised draft for text-based negotiation (2024). Available online at: https://www.twn.my/title2/health.info/2024/hi240304.htm (Accessed May 9, 2026).

[43] World Health Organization. Third meeting of the intergovernmental negotiating body, USA Statement. Available at: https://apps.who.int/gb/inb/e/e_inb-3.html (Accessed May 9, 2026).

[44] World Health Organization. Third meeting of the intergovernmental negotiating body, UK Statement (2022). Available online at: https://apps.who.int/gb/inb/e/e_inb-3.html (Accessed May 9, 2026).

[45] World Health Organization Third meeting of the intergovernmental negotiating body, Australia Statement (2022). Available online at: https://apps.who.int/gb/inb/e/e_inb-3.html (Accessed May 9, 2026).

[46] World Health Organization. Third meeting of the intergovernmental negotiating body, Canada statement (2022). Available online at: https://apps.who.int/gb/inb/e/e_inb-3.html (Accessed May 9, 2026).

[47] World Health Organization. Third meeting of the intergovernmental negotiating body, European Union statement (2022). Available online at: https://apps.who.int/gb/inb/e/e_inb-3.html (Accessed May 9, 2026).

[48] World Health Organization. Third meeting of the intergovernmental negotiating body, Norway Statement (2022). Available online at: https://apps.who.int/gb/inb/e/e_inb-3.html (Accessed May 9, 2026).

[49] Third World Network. WHO: developed countries say they don’t know the meaning of solidarity in INB9 (2024). Available online at: https://www.twn.my/title2/health.info/2024/hi240312.htm (Accessed May 9, 2026).

[50] Third World Network. WHO Pandemic Agreement: a win for multilateralism, a missed opportunity for public health? (2025). Available online at: https://www.twn.my/title2/health.info/2025/hi250502.htm (Accessed May 9, 2026).

[51] Jiang S Kumah E. Strategizing global health governance: unpacking opportunities and challenges for least developed nations within the WHO pandemic treaty framework. Front Public Health. (2023) 11:1321125. doi: 10.3389/fpubh.2023.1321125, 38026386 PMC10657804

[52] Halabi Sam Gostin Lawrence. The new WHO Pandemic Treaty and global health governance: incorporating common but differentiated responsibilities into PABS (2025). Available online at: https://gjia.georgetown.edu/global-governance/the-new-who-pandemic-treaty-and-global-health-governance-incorporating-common-but-differentiated-responsibilities-into-pabs/ (Accessed May 9, 2026).

[53] World Health Organization. Conceptual zero draft for the consideration of the intergovernmental negotiating body at its third meeting (2022). Available online at: https://apps.who.int/gb/inb/pdf_files/inb3/A_INB3_3-en.pdf (Accessed May 9, 2026).

[54] World Health Organization. Zero draft of the WHO CA+ for the consideration of the intergovernmental negotiating body at its fourth meeting (2023). Available online at: https://apps.who.int/gb/inb/pdf_files/inb4/A_INB4_3-en.pdf (Accessed May 9, 2026).

[55] Ramakrishnan Nithin Gopakumar K M. WHO: zero draft of the pandemic instrument creates an “illusion” of equity (2023). Available online at: https://www.twn.my/title2/health.info/2023/hi230207.htm (Accessed May 9, 2026).

[56] Third World Network. WHO: INB bureau to develop another draft negotiating text (2023). Available online at: https://www.twn.my/title2/health.info/2023/hi230902.htm (Accessed May 9, 2026).

[57] Cullinan Kerry. Africa decries weakening of equity provisions in new pandemic accord draft Health Policy Watch (2023). Available online at: https://healthpolicy-watch.news/africa-decries-weakening-of-equity-provisions-in-new-pandemic-accord-draft/ (Accessed May 9, 2026).

[58] World Health Organization. Intergovernmental negotiating body, proposal for negotiating text of the WHO Pandemic Agreement (2023). Available online at: https://apps.who.int/gb/inb/pdf_files/inb7/A_INB7_3-en.pdf (Accessed May 9, 2026).

[59] World Health Organization. Proposal for negotiating text of the WHO Pandemic Agreement (2023). Available online at: https://apps.who.int/gb/inb/pdf_files/inb7/A_INB7_3-en.pdf (Accessed May 9, 2026).

[60] World Health Organization. Revised draft of the negotiating text of the WHO Pandemic Agreement (2024). Available online at: https://apps.who.int/gb/inb/pdf_files/inb9/A_inb9_3-en.pdf (Accessed May 9, 2026).

[61] Schwalbe N Lehtimaki S Hannon E. The pandemictreaty: a forensic review of process and pitfalls. Glob Public Health. (2025) 20:2522916. doi: 10.1080/17441692.2025.2522916, 40587997

[62] Lake SJ Sekalala S. Coercive containment of the Africa Group’s Equity Agenda in the WHO Pandemic Treaty negotiations. Lond Rev Int Law. (2026) 14:lrag009. doi: 10.1093/lril/lrag009

[63] World Health Organization. Proposal for the WHO Pandemic Agreement (2024). Available online at: https://apps.who.int/gb/inb/pdf_files/inb9/A_inb9_3Rev1-en.pdf (Accessed May 9, 2026).

[64] World Health Organization. Intergovernmental negotiating body to draft and negotiate a WHO convention, agreement or other international instrument on pandemic prevention, preparedness and response (2024). Available online at: https://apps.who.int/gb/ebwha/pdf_files/WHA77/A77_10-en.pdf (Accessed May 9, 2026).

[65] Third World Network. WHO: INB bureau refuses to incorporate member states proposals in negotiating text (2023). Available online at: https://www.twn.my/title2/health.info/2023/hi231111.htm (Accessed May 9, 2026).

[66] Parthenay K Godoy P. The WHO pandemic agreement negotiation: introducinga new database (the pandemic agreement database). Glob Policy. (2026) 17:433–45. doi: 10.1111/1758-5899.70140

[67] World Health Organization. Proposed amendments to the International Health Regulations (2005) submitted in accordance with decision WHA75(9) (2022). Available online at: https://apps.who.int/gb/wgihr/pdf_files/wgihr1/WGIHR_Submissions_Original_Languages.pdf (Accessed May 9, 2026).

[68] World Health Organization. Report of the review committee regarding amendments to the International Health Regulations (2005) (2023). Available online at: https://apps.who.int/gb/wgihr/pdf_files/wgihr2/A_WGIHR2_5-en.pdf?sfvrsn=4b549603_12 (Accessed May 9, 2026).

[69] Balakrishnan Vijay Shankar. WHO pandemic treaty: the good, the bad, & the ugly—an interview with Larry Gostin (2023). Available online at: https://healthpolicy-watch.news/who-pandemic-treaty-the-good-the-bad-the-ugly-an-interview-with-larry-gostin/ (Accessed May 9, 2026).

[70] Hamrouni M-O Canal E Alter F. Equity, international cooperation, and global public health: use of the common but differentiated responsibilities principle in the fight against COVID-19. J Int Women Stud. (2022) 23:120.

[71] Third World Network. WHO: pandemic agreement adopted by vote; all eyes on PABS annex (2025). Available online at: https://www.twn.my/title2/health.info/2025/hi250505.htm (Accessed May 9, 2026).

[72] Evaborhene NA. Africa’s critical role in shaping and implementing the pandemic agreement’s PABS annex in an era of fragmentation. Glob Health. (2026) 22:30. doi: 10.1186/s12992-026-01190-3, 41832575 PMC12989124

[73] World Health Organization. Draft PABS annex text (2025). Available online at: https://www.who.int/publications/m/item/a-igwg-3-3-draft-pabs-annex-text (Accessed May 9, 2026).

[74] Jon WJ. Implementation of the WHO pandemic agreement. Bull World Health Organ. (2025) 103:638–40. doi: 10.2471/BLT.25.294146, 41035553 PMC12481221

[75] AVillarreal P Gross A Phelan A. The proposed pandemic agreement: a pivotal moment for global health law. J Law Med Ethics. (2025) 53:56. doi: 10.1017/jme.2025.22 40116223

[76] Third World Network. WHO: member states propose extending PABS negotiations beyond WHA79 (2026). Available online at: https://www.twn.my/title2/health.info/2026/hi260408.htm (Accessed May 9, 2026).

[77] World Health Organization. Draft PABS Annex I.A.2(b).(2025) (2025). Available online at: https://www.who.int/publications/m/item/a-igwg-3-3-draft-pabs-annex-text (Accessed May 9, 2026).

[78] Brahmi P Tyagi V. "Access and benefit sharing mechanism under the multilateral system of the international treaty on plant genetic resources for food and agriculture". In: Laladhas K Nilayangode P Oommen OV, editors. Biodiversity for Sustainable Development. Environmental Challenges and Solutions, vol. 3. Cham: Springer (2017). p. 18–9.

[79] Fidler DP Gostin LO. The WHO pandemic influenza preparedness framework: a milestone in global governance for health. JAMA. (2021) 306:200–1. doi: 10.1001/jama.2011.960, 21750298

[80] World Health Organization. Draft PABS Annex II.C.1 (2025). Available online at: https://www.who.int/publications/m/item/a-igwg-3-3-draft-pabs-annex-text (Accessed May 9, 2026).

[81] World Health Organization. Draft PABS Annex II.B.1 (2025). Available online at: https://www.who.int/publications/m/item/a-igwg-3-3-draft-pabs-annex-text (Accessed May 9, 2026).

[82] World Health Organization. Draft PABS Annex III.A.1 (2025). Available online at: https://www.who.int/publications/m/item/a-igwg-3-3-draft-pabs-annex-text (Accessed May 9, 2026).

[83] World Health Organization. Draft PABS Annex II.C.4 (2025). Available online at: https://www.who.int/publications/m/item/a-igwg-3-3-draft-pabs-annex-text (Accessed May 9, 2026).

[84] World Health Organization. Draft PABS Annex III.A.2(d) (2025). Available online at: https://www.who.int/publications/m/item/a-igwg-3-3-draft-pabs-annex-text (Accessed May 9, 2026).

[85] Rajamani L. Differential Treatment in International Environmental Law. Oxford: Oxford University Press (2006). p. 133.

